# Body Size Variation in a Social Sweat Bee, *Halictus ligatus* (*Halictidae*, *Apoidea*), across Urban Environments

**DOI:** 10.3390/insects12121086

**Published:** 2021-12-03

**Authors:** Rachel A. Brant, Gerardo R. Camilo

**Affiliations:** 1 Department of Biology, University of Missouri, St. Louis 223 Research Bldg, St. Louis, MO 63121, USA; 2 Department of Biology, Saint Louis University, St. Louis, MO 63103, USA; gerardo.camilo@slu.edu

**Keywords:** urban ecology, morphology, bees, functional traits

## Abstract

**Simple Summary:**

Many animal species that dwell in cities have altered aspects of their behavior, morphology, and physiology in order to survive in human-dominated environments. One way in which animals can adapt to survive in novel habitats is by shifting their body size. Body size is an important and flexible trait for insects because the ability to vary body size is linked to better survival and reproduction. In this study, we quantified body size variation in a species of sweat bee and compared the variation between bees residing in three different urban cities. Though studies have assessed urban bee body size previously, this is the first to compare bees from different cities. Similar to the human experience, no two cities are alike for bees. Therefore, we predicted that bees would show differences in the spread of body size in order to adapt to each unique city. We found that bees in three different environments all showed high variation in body size, but that the variation differed depending on location. This study is one of the first multi-city studies, and this is a trend we hope continues as urban research advances.

**Abstract:**

High morphological variation is often associated with species longevity, and it is hypothesized that urban-dwelling species may require more plasticity in functional traits such as body size in order to maximize fitness in heterogeneous environments. There has been published research regarding the functional trait diversity of urban bee pollinators. However, no two cities are identical, so the implementation of multi-city studies is vital. Therefore, we compared body size variation in female *Halicus ligatus* sweat bees from May–October 2016 from three distinct Midwestern United States cities: Chicago, Detroit, and Saint Louis. Additionally, to elucidate potentially influential environmental factors, we assessed the relationship between temperature and measured body size. We collected bees in community gardens and urban farms and measured their head width and intertegular distance as a proxy for overall body size. We utilized an ANCOVA to determine whether body size variation differed significantly across the three surveyed cities. Results indicated that *H. ligatus* females in Chicago, Detroit, and Saint Louis had significantly different body size ranges. These findings highlight the importance of intraspecific body size variation and support our prediction that bees from different urban environments will have distinct ranges in body size due to local ecological factors affecting their populations. Additionally, we found a significant influence of temperature, though this is probably not the only important ecological characteristic impacting bee body size. Therefore, we also provided a list of predictions for the future study of specific variables that are likely to impact functional trait diversity in urban bees.

## 1. Introduction

Increased urbanization is rapidly altering pristine landscapes and the habitats of numerous species. Though land use change is considered one of the greatest threats to animal conservation [1], many organisms are thriving in cities by adapting aspects of their life history [2], behavior [3], phenology [4], and morphology [5]. Cities boast high bee species richness and abundance [6,7,8,9,10], and research indicates that abiotic, biotic, and anthropogenic factors such impervious surfaces [11], human population density [12], and land management practices [8], can impact bee community composition. There has been a large influx of research devoted to documenting bee populations in cities; however, the majority of published work focuses solely on quantifying alpha diversity. Critical adaptations and plasticity responses to urbanization remain poorly understood in most urban bee species.

One way that organisms can cope with novel environments is by shifting functional traits. Functional traits are strongly influenced by local environmental factors [5,13] and have implications for individual fitness and overall species persistence. Only a handful of studies have evaluated plasticity in functional traits of urban-dwelling bees. For example, Eggenberger et al. [5] measured morphological features of bumblebees to determine how functional trait diversity differs between urban- and rural-dwelling bees. Their results indicated that city bees exhibited higher functional trait diversity compared to conspecifics living in rural systems [5]. Similarly, in a study by Bucholz and Egerer [14], bumblebees residing in urban areas showed higher variation in body size compared to those found in natural habitats [14]. These studies provide strong evidence that plasticity in functional traits, and particularly in body size, enables bees to persist in highly heterogeneous, urban landscapes. However, almost all published studies regarding bee body size variation have focused on *Bombus* species in a single metropolitan area. For this reason, there remains a deficiency in our understanding of non-*Apidae* species, which are equally if not more abundant in many cities [15]. Additionally, single-city studies, though informative for local conservation efforts, cannot reveal any significant broad ecological patterns impacting bees across a diverse range of urban areas [16].

Therefore, the goal of our study was to quantify and compare body size variation in a facultatively eusocial sweat bee species, *Halictus ligatus*, in three distinct Midwestern cities and to determine whether patterns of body size variation were conserved across urban landscapes or diverged among cities. Additionally, we aimed to elucidate the potential influence of climatic factors on documented body size. We chose to assess body size variation because it is a highly adaptable functional trait associated with numerous important functions including dispersal ability [17], pollination efficiency [18], longevity [19], and population persistence [20]. For example, bumblebee species that demonstrate high intraspecific body size variation are less susceptible to population decline [21]. Additionally, adult body size is directly correlated with foraging distance in bees, with larger body sizes allowing for longer foraging flights [17]. Therefore, variation in body size in bees, though phylogenetically constrained, may help lessen the negative impacts of patchy, unreliable urban environments.

We chose the sweat bee, *Halictus ligatus*, as our focal species for two reasons. Firstly, research indicates that *H. ligatus* exhibits plasticity in intraspecific body size as a result of environmental fluctuations [22,23], making this species an excellent candidate for the scope of our study. Secondly, *H. ligatus* is one of the most common bee species in many cities in North America [10,15,24] but is rarely incorporated into targeted urban bee research. Because of our emphasis on multi-city research, it was imperative to utilize a highly ubiquitous species capable of thriving in a variety of urban environments.

In this study, we first asked whether the body size variation in *H. ligatus* followed similar patterns in three cities in the Midwestern United States:Chicago, Detroit and Saint Louis. We predicted that female *H. ligatus* sweat bees residing in Chicago, Detroit, and Saint Louis would all exhibit high variation in body size, as this is often linked to higher fitness and reproductive success [20,21]. Secondly, we asked whether observed body size variation differed between the surveyed populations from these different urban areas. We predicted that the overall spreads of documented body size of *H. ligatus* bees sampled from the three surveyed cities would be significantly different from one another, due to unique selection pressures and ecological factors that inevitably vary among cities. Thirdly, in an effort to elucidate potential effects on any observed variation, we asked how climate may be influencing body size variation in *H. ligatus*. We predicted that seasonal fluctuations in temperature and cumulative growing degree days would significantly affect body size variation in *H. ligatus* sweat bees in cities. Though numerous ecological factors can impact insect body size, there is ample research regarding the important role of temperature on development [25,26], survival [27], and adult body size in insects [25]. For example, growth rate and adult body size have nearly linear relationships with temperature [25], and previous research shows that the cumulative growing degree days (CGDD) is a significant predictor of ectotherm developmental rate [28]. Therefore, we predicted that climate would have a significant relationship with body size in *H. ligatus* sweat bees.

## 2. Materials and Methods

### 2.1. Survey Methods

**Figure 1:**
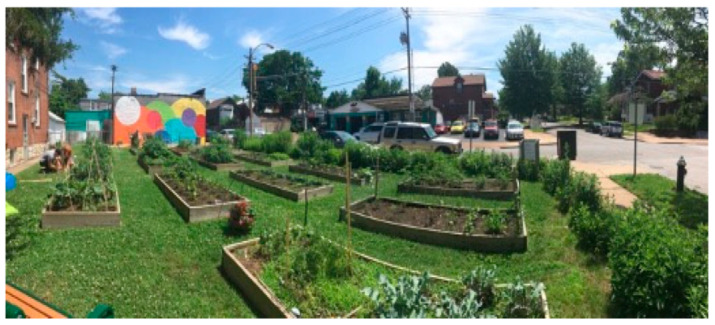
Example of one of the included community garden sites in Saint Louis. All of the survey sites were urban community gardens which consist primarily of raised beds and rows of produce maintained by volunteers and community members.

We conducted bee surveys in the urban core of three densely populated Midwestern United States cities: Saint Louis, Missouri, Detroit, Michigan, and Chicago, Illinois. We surveyed 24 community gardens of relatively similar size in all three cities (Figure 1). Our study included 7 community gardens in Chicago, 7 in Detroit, and 10 in Saint Louis. All gardens were located in previously vacant city lots. All gardens contained both fruit and vegetable plants consumed by nearby neighbors, as well as native flowering species (e.g., *Echinacea purpurea*).

We surveyed each garden from 10am–1pm every two weeks, from April to October of 2016. We used two common bee collection methods: hand netting and bowl trapping. Hand netting involved researchers utilizing large aerial nets to collect *H. ligatus* females at each sample site. Hand netting occurred at a rate of one person per hectare per 0.25 h. Caught bees were then placed in ethyl acetate insect jars for preservation. For bowl traps we utilized blue and yellow plastic bowls 10 cm in diameter and filled each bowl with a solution of soapy water to break the surface tension. We placedbowls throughout each garden site at a distance of every 3 m. After 24 h, we retrieved the bowls and sorted all the insects. Any submerged *H. ligatus* females were dried, pinned, labeled, and included in our study. All specimens were pinned, labeled, and deposited in the insect collection at Saint Louis University.

### 2.2. Bee Body Size Measurements

**Figure 2:**
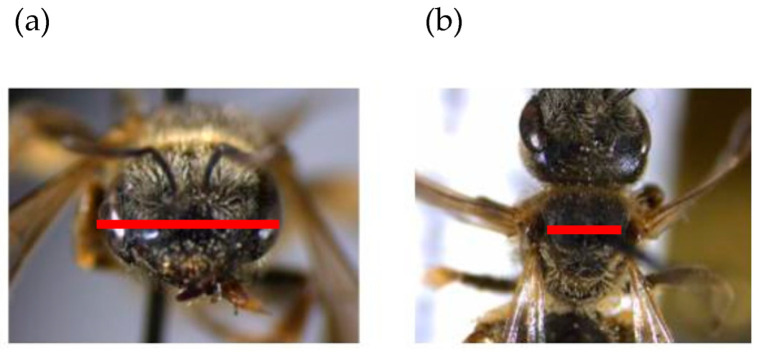
Sample measurement of (**a**) head width and (**b**) intertegular distance of a female *H. ligatus*.

We measured both head width and the intertegular distance as a proxy for overall body size of 240 *H. ligatus* females. Head width has been examined extensively by researchers as an indicator of caste in eusocial insects, and correlates strongly with overall body size [22] (Figure 2a). Intertegular distance is defined as the space between wing joints and has been shown to be an indicator of resource availability for larvae and signal foraging range in adults [17] (Figure 2b). Though each measurement is associated with slightly different information, head width and intertegular distance are tightly correlated (r^2^ = 0.96; Figure 2). Each individual female was measured twice for accuracy, using the average as the final intertegular measurement. Body measurements were taken using a Leica S6D stereomicroscope with the LAS microscope imaging software version 4.6. To simplify all subsequent statistical analyses, we used solely the head width as a proxy for *H. ligatus* body size.

### 2.3. Statistical Analyses

We used an analysis of covariance (ANCOVA) to ascertain the quantitative relationship between intertegular distance and head width. An ANCOVA compares the slope of the regression line of head width and intertegular distance for each city, allowing us to compare the overall variation in body size of three urban populations. If significant differences were detected, we then used a Tukey–Kramer HSD a posteriori test to determine where those differences lay.

To determine whether differences in climate influenced the observed differences in body size variation, we first extracted daily maximum, daily minimum, and daily average atmospheric temperatures for each day from 1 January 2015–31 December 2016 for each city. All climatic data were downloaded from the NOAA NCDC climate database for Chicago, Detroit, and Saint Louis. [29]. Using extracted temperature data, we calculated growing degree days (base 10 °C beginning January 1). We then implemented a one-way ANOVA to determine whether temperature and CGDD varied significantly throughout the year in Chicago, Detroit, and Saint Louis. If significant differences were detected, we then used a Tukey–Kramer HSD a posteriori test to determine where those differences lay. Because daily maximum air temperature, daily minimum air temperature, and cumulative growing degree days are all highly correlated with the month (r^2^ = 0.87, r^2^ = 0.88, and r^2^ = 0.96, respectively),we used “month” as a proxy for overall climate in subsequent analyses. If we found significant differences in climate, we then utilized a linear model with head width as the response variable and “month” and “city” as interacting fixed effects. Due to the small sample size in the month of October, we combined data collected in both September and October, renaming this set “SepOct”. All statistical analyses were conducted using R studio v.1.3.1093.

## 3. Results

### 3.1. Does Body Size Variation Differ among Midwestern Cities?

Between the months of May and October of 2016 we collected a total of 240 *Halictus ligatus* females across all three cities: 73 individuals from Chicago, 50 from Detroit, and 117 individuals from Saint Louis. The following are the total individuals sampled by month from Chicago, Detroit, and Saint Louis, respectively: May (4, 29, 6), June (27, 4, 36), July (8, 8, 32), August (25, 7, 37), and SeptOct (9, 2, 6).

**Figure 3:**
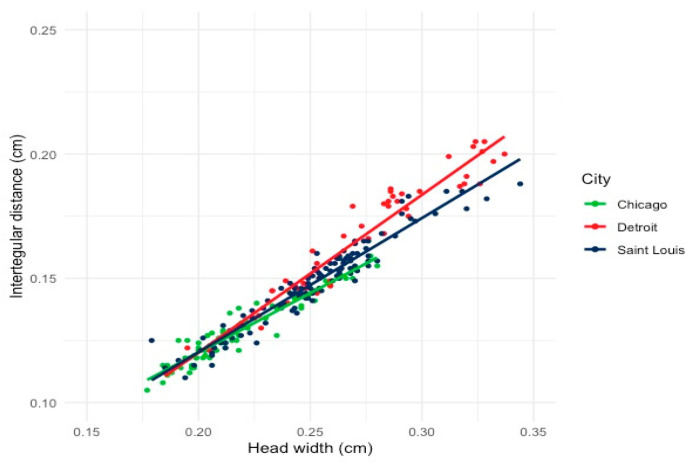
Variation in body size of *H. ligatus* females from three Midwestern cities: Chicago, Detroit, and St. Louis. The spread of body size variation was greatest in Detroit. Additionally, the bee with the largest head width was located in Saint Louis (0.337 cm) and the bee with the smallest head width was sampled from Chicago (0.177 cm).

The results of the ANCOVA test, indicated that body size variation was significantly different across the three surveyed urban cities (Figure 3). To compare the degree of body size variation between *H. ligatus* bees in Chicago and Detroit and between Detroit and Saint Louis, we performed a Tukey HSD post hoc analysis on the previous ANCOVA test. The results of the Tukey HSD test indicated that all three cities showed significantly different variation in bee body size (Table 1).

Chicago had the lowest degree of body size variation, with the largest individual measured at 0.280 cm and the smallest individual at 0.177 cm. Detroit had the largest spread in variation of body size among the sampled *H. ligatus* females (largest = 0.337 cm and smallest = 0.185 cm). This finding supports previous studies suggesting that body size variation in a population aids the persistence of urban-dwelling *H. ligatus* populations. However, the significant differences in the slope of each regression line suggest that the extent of bee body size variation is likely to be shaped by local environmental pressures.

### 3.2. Do differences in Climate Influence Differences in Body Size Variation?

**Figure 4:**
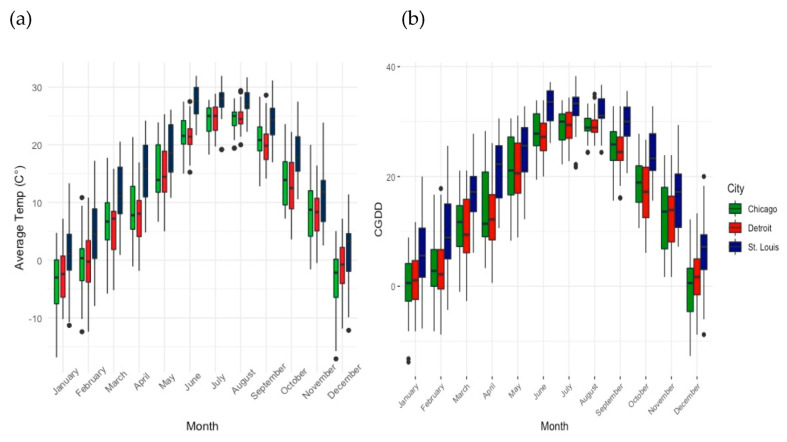
Climatic differences between the three sampled cities, Chicago, Detroit, and Saint Louis, including (**a**) average temperature from NOAA climate data throughout 2016 and (**b**) cumulative growing degree days throughout 2016.

When assessing the role of climate on bee body size variation, we first determined whether there were significant differences in temperature and cumulative growing degree days throughout 2015 and 2016 between the three surveyed cities. The results of both ANOVA tests indicated significant differences in both daily temperature and cumulative growing degree days. After performing Tukey HSD post hoc analyses on the previous ANOVAs, the results showed that Saint Louis had a significantly different temperature and CGDD throughout the year compared to both Detroit and Chicago, but Detroit and Chicago were not significantly different from each other (Table 2 and Table 3). Figure 4 illustrates the variation in temperature and CGDD in 2016 between the three surveyed cities.

Because the Tukey HSD post hoc analyses revealed significant differences in temperature and cumulative growing degree days, we proceeded with a linear model to determine whether climatic variation in each city was contributing to the observed head width variation of *H. ligatus* bees. The results of the linear model indicated that the variation in climate in each city did influence the head width range (Table 4).

**Figure 5:**
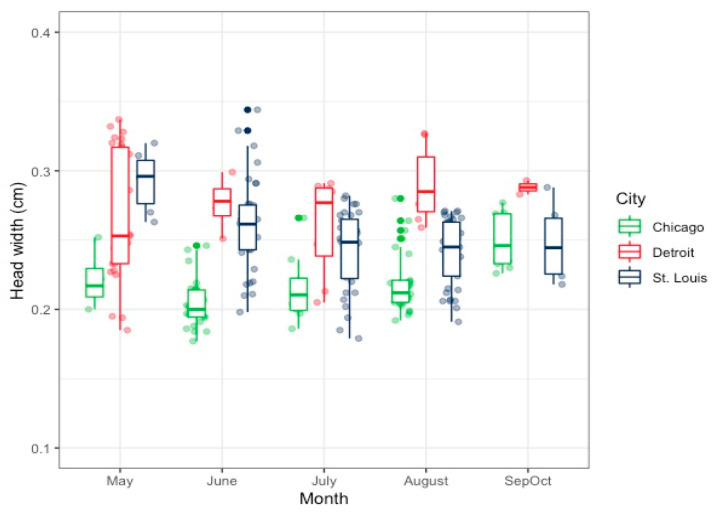
Variation in body size of female *Halictus ligatus* sweat bees throughout the season in Chicago, Detroit, and Saint Louis.

Specifically, bee head width in May and June in Saint Louis varied significantly from bees measured in Chicago and Detroit during the same time period (*p* = 0.02 and *p* = 1.12 × 10^−3^, respectively). Additionally, bees surveyed and measured during the fall season (SepOct) had significantly different head widths compared to bees surveyed in all other months (*p* = 0.01). This variation among head widths for bees from the three surveyed cities is illustrated in Figure 5.

## 4. Discussion

In this study we tested whether the social sweat bee, *Halictus ligatus*, exhibited similar body size variation in three geographically separated urban cities in the Midwestern United States. The overall findings indicated that urban-dwelling *Halictus ligatus* exhibited variation in body size in- Chicago, Detroit, and Saint Louis. We hypothesized that urban social sweat bees would exhibit high variation in body size, as this is often indicative of population persistence and task specialization in social insects [30]. We found that body size variation was greatest in Detroit and least in Chicago. We hypothesized that the observed body size variation would diverge among cities (Figure 3) due to the unique aspects of each urban region shaping the selection pressures. These findings are not unique to bee functional trait diversity. In a recently published article, the authors reviewed numerous studies on urban bee community composition and concluded that the findings in the current literature remain inconclusive regarding the benefit or detriment of urbanization to bee species diversity [6]. Additionally, they concluded that the characteristics influencing populations varied greatly across cities, with some studies reporting floral abundance as the best predictor of bee populations [31], while others stated impervious surfaces were the most influential [32]. The results from this review align with our findings and our argument that no two urban regions are identical. Additionally, it is understood that body size variation is an extremely important trait for insect persistence, fitness, and survival. However, in urban agricultural settings with diverse floral resources [33], bee body size variation is vital. Bee size is correlated with flight distance [17], task specialization, and floral handling behavior [34]. Having a range of body sizes in bee populations not only provides insurance for their own persistence [20] but aids in the ecosystem service humans rely on, i.e., pollination.

To begin to elucidate potential environmental drivers of the observed body size variation in *H. ligatus* sweat bees, we used the month to explore the relationship between each city’s climate and the body size range. The results indicated that temperature and CGDD in Saint Louis varied significantly from those in Chicago and Detroit, but the temperature and CGDD in Chicago and Detroit did not significantly differ from one another. This aligns with our prediction that, among the three cities, there would be differences in seasonality. However, the lack of significant differences in climate between Chicago and Detroit is likely driven by their similar latitudes (these cities are located at 41.8781° N, 87.6298° W and 42.3314° N, 83.0458° W, respectively). When assessing the importance of the temperature patterns of each city on the observed head width variation, the results supported our prediction that unique attributes of each surveyed city, such as climate and seasonality, would explain differences in the range of body sizes of *H. ligatus* bees. Previous research suggests that rainfall, temperature, and length of the growing season can influence development and overall adult body size of *H.ligatus* [22,23], and our results align with these findings. However, few studies have compared functional traits of bees in urban regions, and those that have typically assess only one city. Urban ecology has been criticized for lacking repeated studies across cities to account for differences in the urban experience [16]. Our study is the first to compare bee body size variation in three distinct urban systems and assess the role of seasonality and temperature on body size variation in urban-dwelling bees. We found that body size variation, an important trait for insects’ survival and reproduction [20], is highly conserved in urban areas. However, the range of body sizes differs among cities, driven at least in part by climate.

Due to the vast number of characteristics that are likely to impact urban bee functional trait diversity, we cannot at this time state that climate is the main factor driving the patterns revealed in this study. The literature highlights the importance of numerous mechanisms, including mechanisms related to floral diversity, queen size, colony size, and even chemical exposure, for affecting insect body size. Though the aforementioned environmental aspects were out of the scope of our study, we provide three predictions for future research in the hope of provoking ideas for future studies regarding urban bee morphological variation.

Prediction 1: Queen size directly influences worker body size.

We know from research on eusocial bee species, including *Halictus ligatus*, that worker body size is highly correlated with queen size, regardless of environmental conditions [22,23]. Therefore, we predict that both average worker body size and overall variation of body size will be significantly influenced by queen size. However, because *H. ligatus* are plastically eusocial, and sociality is dependent upon environmental factors [22,23,35], the relationship between queen size, worker body size, and the environment is highly complex. It is likely that body size variation is correlated with an entirely separate suite of factors when *H. ligatus* are solitary or aggregated. Like a pendulum, the overall importance of queen size or environment may alter yearly as their sociality fluctuates.

Prediction 2: Resource availability and provisioning can either increase or decrease bee body size variation, which is likely to vary by site

We know that diet breadth is one of the largest determining factors of insect body size [36]. For example, bumblebee body size has been shown to be highly influenced by food resources and position in the nest [37,38]. Additionally, *Bombus terricola* fed at differing rates across colonies showed significant differences in average body size over time [37]. Floral resources in the wild are not constant, and the rewards available to insects fluctuate depending upon phenology, competition, and seasonality. Therefore, we predict that resource availability throughout the season and across sites correlates with body size variation in urban-dwelling *H. ligatus*. Assessing not only floral diversity and abundance of habitats but also pollen lipid, and amino acid content in the wild can provide important insights into the nutrients available to bee populations and how this may influence the bee body size variation in urban environments. Additionally, using the marginal value theorem [38] to predict the patch-leaving dynamics of bees based upon floral resources and body size could provide the data necessary to understand the relationship between floral landscape and bee functional trait diversity.

Prediction 3: Chemical aspects of the urban environment may impact both plants and pollinators.

Cities have high atmospheric CO_2_ [39], and research has been performed on several aspects related to anthropogenically modified environments, including heightened atmospheric carbon dioxide, functional traits [40], cognitive ability [41], and overall survival [42,43]. For example, elevated atmospheric CO_2_ has been shown to decrease insect cognitive functioning in bees, prolong development in locusts, and increase endogenous immune responses in monarch butterfly larvae [44]. Additionally, heightened CO_2_ is linked to nutrient dilution in plants, due to trade-offs between growth and nutrient acquisition [45,46]. This phenomenon, known as the nutrient dilution hypothesis, is likely to have implications for resource provisioning, larval development, and overall adult body size in bees. The relationship between bee body size variation and atmospheric CO_2_ has yet to be assessed. However, based upon the aforementioned work, we predict that functional trait diversity, particularly in a trait as plastic as body size, will likleydecrease as a result of both diluted nutrients in pollen provisioning and prolonged development. Extracting and analyzing the pollen nutrient content from plants in urban spaces, as well as measuring the overall body size variation of bees residing in natural areas and in cities with known elevated CO_2_ levels (e.g., Phoenix [47]) would elucidate how CO_2_ may affect both plants and insects in urban regions.

## 5. Conclusions

The need for holistic understanding of urban bee populations is undisputed. Research on bee abundance and diversity is often given priority when attempting to determine an urban region’s potential for pollination services, with few studies assessing aspects of phenology, functional trait diversity, behavior, or genetics. It is understood that variation in body size in social bees can provide a protective “buffer”, allow individuals to specialize in certain tasks, and aid in maintaining colony fitness. However, functional trait diversity remains poorly understood in urban-dwelling bees. This study is the first to assess variation in body size in a non-*Apidae* species residing in three distinct urban environments. Though methodologically tedious, we argue that it is imperative to produce multi-city analyses and incorporate lesser-known bee species in order to broaden research findings that can promote urban conservation policy more holistically.

## Figures and Tables

**Table 1:** Results of a Tukey HSD post hoc analysis reveal significant differences between the variation of body size of *Halictus ligatus* females in Detroit, Chicago, and Saint Louis.

Cities	Diff	Lwr	Upr	*p*-Value
Detroit-Chicago	0.05	0.04	0.07	<1.0 × 10^−4^
St. Louis-Chicago	0.034	0.02	0.05	<1.0 × 10^−4^
St. Louis-Detroit	−0.02	−0.03	−7.0 × 10^−3^	9.0 × 10^−4^

**Table 2:** Results of a Tukey HSD post hoc analysis reveal significant differences in the average temperature in 2016 between St. Louis and Chicago and between St. Louis and Detroit.

Cities	Diff	Lwr	Upr	*p*-Value
Detroit-Chicago	0.059	−1.75	1.86	0.99
St. Louis-Chicago	4.10	2.30	5.91	3.00 × 10^−6^
St. Louis-Detroit	4.05	2.24	5.85	5.00 × 10^−6^

**Table 3:** Results of a Tukey HSD post hoc analysis reveal significant differences in the average cumulative growing degree days in 2016 between St. Louis and Chicago and between St. Louis and Detroit.

Cities	Diff	Lwr	Upr	*p*-Value
Detroit-Chicago	−30.62	−194.53	133.30	0.90
St. Louis-Chicago	465.37	301.45	629.29	1.00 × 10^−10^
St. Louis-Detroit	495.99	332.07	659.90	1.00 × 10^−10^

**Table 4:** The results of the linear model with head width as the response variable and month and city as interacting fixed effects indicate significant relationships between bee head with and city climate.

Coefficients:	Estimate	Std. Error	*t* Value	Pr(>|t|)
(Intercept)	0.22	5.83 × 10^−3^	37.73	<2.00 × 10^−16^
Month July	−4.54 × 10^−3^	0.01	−0.38	0.70
Month June	−0.02	8.09 × 10^−3^	−1.93	0.05
Month May	1.46 × 10^−3^	0.02	0.09	0.93
Month Sep Oct	0.03	0.01	2.50	0.01
City Detroit	0.07	0.01	5.63	0.00
City Saint. Louis	0.02	7.55 × 10^−3^	2.87	0.00
Month July: City Detroit	−0.02	0.02	−1.30	0.19
Month June: City Detroit	1.85 × 10^−3^	0.02	0.09	0.93
Month May: City Detroit	−0.03	0.02	−1.31	0.19
Month Sep Oct: City Detroit	−0.03	0.03	−1.18	0.24
Month July: City Saint. Louis	5.83 × 10^−3^	0.01	0.42	0.67
Month June: City Saint. Louis	0.03	0.01	3.29	1.12 × 10^−3^
Month May: City Saint. Louis	0.05	0.02	2.44	0.02
Month Sep Oct: City Saint. Louis	−0.02	0.02	−1.29	0.20

## Data Availability

The data presented in this study are available in the Supplementary Material.

## Supplementary Materials

The following are available online at https://www.mdpi.com/article/10.3390/insects12121086/s1, Data: manuscript_supplementary.csv.
